# Genotypic Profiling of *Bacillus cereus* Recovered from Some Retail Foods in Ogun State, Nigeria, and Their Phylogenetic Relationship

**DOI:** 10.1155/2020/3750948

**Published:** 2020-09-14

**Authors:** Titilayo O. Adesetan, Moses O. Efuntoye, Olubukola O. Babalola

**Affiliations:** ^1^Department of Microbiology, Olabisi Onabanjo University, PMB 2002, Ago-Iwoye, Ogun State, Nigeria; ^2^Food Security and Safety Niche, Faculty of Natural and Agricultural Sciences, North-West University, Private Bag X2046, Mmabatho 2735, South Africa

## Abstract

Identifying *Bacillus cereus* with conventional methods is neither specific nor rapid because of the close relatedness of the *B. cereus* group, hence the need for molecular methods. Genotypic profiling of *B. cereus* isolates from food was obtained by Random Amplified Polymorphic DNA-polymerase chain reaction (RAPD-PCR) using OPR13 primer. A dendrogram was drawn with the Numerical Taxonomy System of Statistic (NTSYS) software. Thirty of the isolates were subjected to molecular identification by 16S rDNA sequencing. The thirty sequences were deposited in GenBank for accession number. Phylogenetic relationship of the 16S rDNA sequence obtained was carried out with the Multiple Alignment using Fast Fourier Transform (MAFFT) software version 7.0. The evolutionary tree was drawn using the Molecular Evolutionary Genetics Analysis (MEGA 6) software. The dendrogram generated for the RAPD profile showed that all the strains are closely related, with a similarity coefficient of 70%. The isolates were confirmed with 16S rDNA sequencing as *B. cereus.* The thirty sequences deposited in GenBank were given accession numbers: KX574760–KX574769, KX610811–KX610820, MT757957-MT757963, and MT772282-MT772284. By comparing the phylogenetic relationship, eleven of the strains did not cluster with the reference strains from the GenBank but form distinct clades, which means they are likely to be of different ancestors. Conventional methods rarely differentiate bacteria of the same species into clade, neither can it describe their ancestral lineage. Therefore, it is important to employ molecular methods in identifying bacteria to give detailed information about them.

## 1. Introduction

*Bacillus cereus* is Gram-positive rod, motile with peritrichous flagella, and ubiquitous. It can multiply in soil [1] and has also been found in food [2–4]. It survives adverse conditions through endospore formation [2, 5]. Spores are more resistant to dry heat and radiation than vegetative cells, and resistance to heat is more considerable in reduced water activity food [6]. The *B. cereus* group is now made up of seven members [7] in which *B. cereus*, *B. anthracis,* and *B. thuringiensis* are the most significant and are closely related [8]. Therefore, identifying members of the *B. cereus* group with biochemical or physiological characteristics is difficult because these characteristics complicate their accurate peculiarity [9]. Biochemical tests do not give distinct differentiation among the groups, but molecular techniques provide fast and accurate identification of the microorganisms [10]. Desai and Varadaraj [3] used polymerase chain reaction (PCR) to confirm twelve isolates as *B. cereus* out of twenty-six isolates previously characterized as *B. cereus* with the conventional method.

Molecular methods such as Random Amplified Polymorphic DNA (RAPD) [11], Multilocus Enzyme Electrophoresis (MLEE) [12], Amplified Fragment Length Polymorphism [13], and Multilocus Sequence Typing (MLST) [14–16] have been found useful in the study of *B. cereus*.

RAPD, which is a form of PCR, is used to study the genetic diversity of an individual. The short RAPD primers of 10 nucleotides are used to amplify the random sequences of the targeted DNA with low temperature during the annealing stage. It is a widely used tool in the study of genetic diversity, population and evolutionary genetics, animal-plant-microbe relationships, plant and animal breeding, pesticide/herbicide resistance, and forensic studies [17–19].

Phylogenetics has made it possible to study the evolutionary history and associations that exist among individuals and groups of organisms, e.g., species, population, or genes. Phylogenetic inference is used to ascertain such association, and the outcome is represented with a phylogenetic tree. The three main inference methods for deducing molecular phylogenies are maximum parsimony, maximum likelihood, and pairwise distances [20]. Phylogeny is presently used in many disciplines such as molecular biology, epidemiology, genetics, ecology, conservation biology, evolution, and forensics.

In Nigeria, foods are retailed by people with little or no knowledge about personal hygiene. Vegetables are sometimes put on the bare floor and handled with bare hands before being sold to consumers. Cooked foods like rice and spaghetti sometimes had contact with the hands of the seller when the spoons and fork used in taking the food are supported with bare hands that have been used in handling money laden with pathogenic bacteria. Smoked fish and hides are exposed to dust, flies and sold with bare hands to consumers. Fried meats are sold inside old newspapers, which might have been contaminated. Knowing fully well that *B. cereus* has been described as a volatile human pathogen, there is need to confirm the isolates recovered from these retailed foods as *B. cereus* using 16S rDNA sequencing, to carry out genotypic profiling of the isolates to confirm if strains in retailed foods are the same, and to determine the relationship of some of the genes with closely related genes in GenBank.

## 2. Materials and Methods

### 2.1. Extraction of Genomic DNA

One hundred *B. cereus* isolates from some retailed foods previously identified with biochemical tests were randomly selected for further confirmation with molecular methods. Each bacterial isolate was subcultured on Luria-Bertani Agar (Merck, South Africa) and incubated at 37°C for 18–24 hours. Genomic DNA was extracted using the Zymo Soil DNA kit (Zymo Research, USA) by following the manufacturer's instructions written in the manual.

### 2.2. Primers Employed in This Study

The primers employed were synthesized by IDT (Integrated DNA Technology, USA). The primers are F1/R2 (F1-5′- AGA GTT TGA TCI TGG CTC AG–3'; R2 5′-ACG GIT ACC TTG TTA CGA CTT–3′) [21] used for 16S rDNA identification while OPR 13 (5′- GGA CGA CAA G–3′) [22] was used for genetic profiling. The primers were reconstituted with Tris/EDTA buffer according to the manufacturer's instruction.

### 2.3. Genetic Typing of *Bacillus cereus* with RAPD-PCR

The OPR13 primer was used for the amplification of random segments of the genomic DNA of *Bacillus* strains. The RAPD-PCR assay was performed in a reaction volume of 25 *μ*l containing 12.5 *μ*l Master mix, 11 *μ*l nuclease-free water, 0.5 *μ*l primer, and 1 *μ*l DNA template. The PCR cycles consisted of an initial denaturation step at 94°C for 4 min, then 40 cycles comprising DNA denaturation at 94°C for 1 min, primer annealing at 35°C for 1 min, and DNA extension at 72°C for 2 min, then a final extension step at 72°C for 5 min [23]. RAPD-PCR bands were separated by electrophoresis in a 2.0% (w/v) agarose gel (LASEC) containing SYBR safe stain (Life Technologies, Thermofisher), immersed in Tris/Acetic acid/EDTA (TAE) (BioRad, USA) buffer, run at 80 V for 90 min. Molecular Ruler (Thermofisher) 1kb base pairs (bp) ladder was used as a molecular weight marker. After the migration of DNA bands, the gel was photographed on the Gel Doc 2000 Image analyzer (BioRad, USA).

### 2.4. Analysis of RAPD-PCR Profile

Each gel was examined, and the presence or absence of polymorphic bands in individual lanes was scored 1 and 0, respectively. The scored bands were subjected to the Numerical Taxonomy System of Statistic (NTSYS) software. In NTSYS analysis, the scatter diagram of the scored bands is useful in revealing a grouping.

### 2.5. 16S rDNA Identification

The primer set F1/R2 was used for the identification. They were performed in reaction mixtures containing 25 *μ*l Master mix, 22 *μ*l nuclease-free water, 1.0 *μ*l primer, and 2 *μ*l template DNA making a final volume of 50 *μ*l. The PCR amplification was performed in a thermal cycler (C1000 Touch, BioRad) with the following conditions: initial denaturation at 96°C for 5 minutes, followed by 30 cycles at 96°C for 45 seconds, annealing at 56°C for 30 seconds, extension at 72°C for 2 minutes, and a final extension at 72°C for 5 minutes. The PCR product was analyzed and viewed as described above.

### 2.6. Sequencing of the PCR Product

Thirty PCR products from 16S rDNA were submitted to Inqaba Biotechnical Industrial (Pty) Ltd, Pretoria, South Africa, for sequencing. The PCR products were sequenced in the forward and reverse directions on the ABI PRISM™ 3500xl Genetic Analyser. Purified sequencing products were analyzed using CLC Main Workbench 7 followed by a BLAST search in the National Centre for Biotechnology Information (NCBI). The thirty sequences were deposited in GenBank for accession number.

### 2.7. Phylogenetic Analysis

16S rDNA gene of the *B. cereus* was also used for the phylogenetic analyses in order to establish the relationship among them. The search for possible reference nucleotide sequences in the NCBI GenBank database was achieved by using the partial sequences from the 16S rDNA of *Bacillus cereus* [24]. Multiple alignments of nucleotide sequences were obtained with the software Multiple Alignment using Fast Fourier Transform (MAFFT) version 7.0 [25], while two main methods distance-based and character-based were employed in drawing the tree using Molecular Evolutionary Genetics Analysis 6 (MEGA 6) [26].

## 3. Results

### 3.1. Genetic Profiling

**Figure 1:**
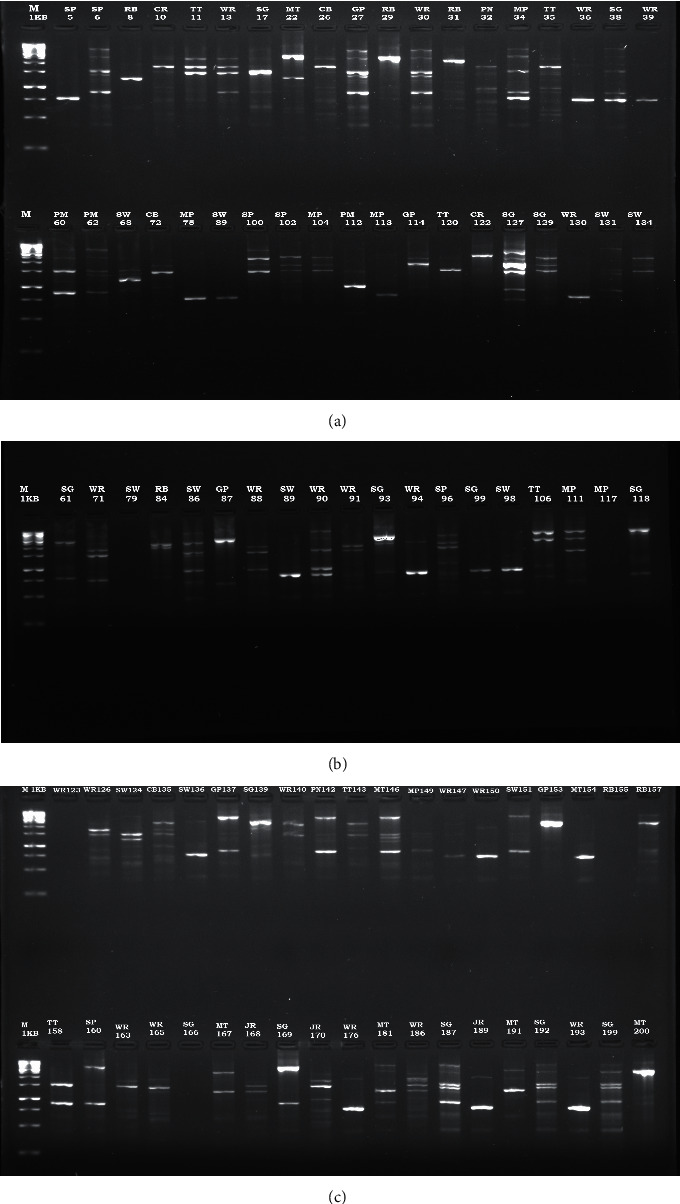
Agarose gel electrophoresis for RAPD-PCR profiles obtained with primer OPR13 for strains in the *B. cereus* group. M: marker, WR: white rice, JR: jollof rice, TT: smoked titus, GP: green pea, SG: spaghetti, RB: runner bean, MP: meat pie, CR: carrot, MT: meat, CB: cabbage, SW: smoked African chad, SP: sweet pepper, and PN: smoked blue whiting.

**Figure 2:**
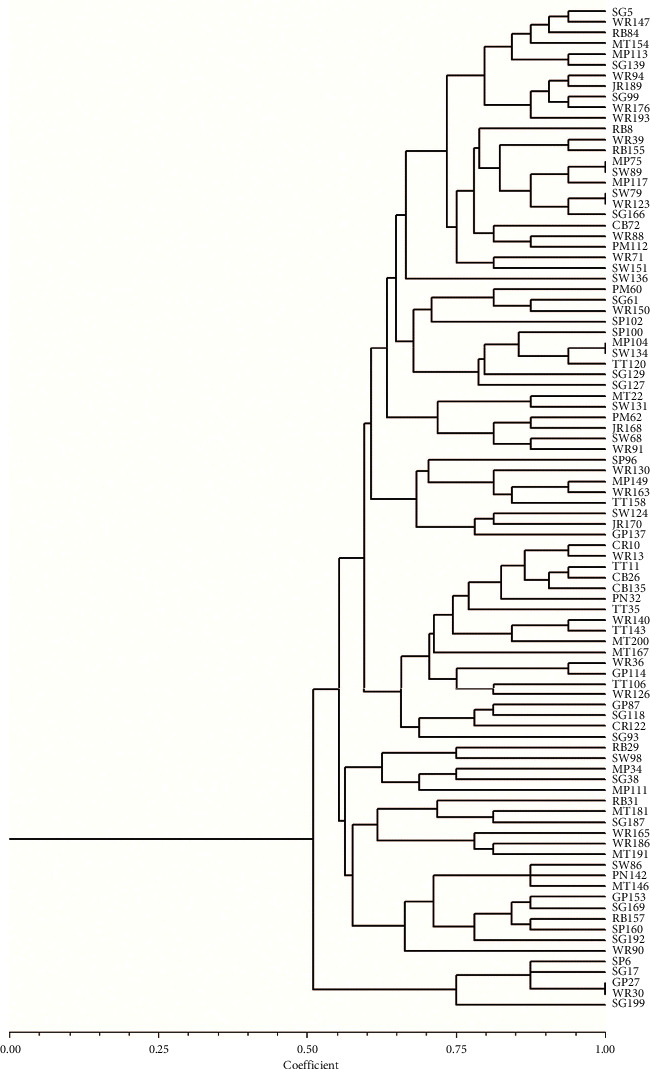
Dendrogram from computer-assisted analysis of RAPD-PCR profiles obtained for *Bacillus cereus* isolates.

Results from Agarose gel electrophoresis using Randomly Amplified Polymorphic DNA-PCR (RAPD-PCR) for the genetic profiling of the isolates showed that many of the isolates expressed multiple bands characteristic of RAPD-PCR (Figures 1(a)–1(c)). Some showed a single band while others did not show any band at all. Some of the isolates on the lanes SP_5_, WR_36_, WR_39_, MP_75_, SW_89_, MP_113_, WR_130_, SW_136_, WR_150_, MT_154_, WR_176_, JR_189_, WR_193_, SW_89_, SG_99,_ and SW_98_showed pronounced single bands with an amplified fragment of about 750 bp (Figure 1). The dendrogram generated from computer analysis of the RAPD-PCR using the NTSYS software revealed that all the bacteria are closely related while those grouped together, that is, MP_75_ and SW_89_, only showed single band and SW_79_ and WR_123_ did not produce any band while GP_27_ and WR _30_, MP_104_ and SW_134_ are the same strain (Figure 2). The OPR13 primer used for the genetic profiling gave a good representation. Many of the isolates showed multiple bands characteristic of RAPD-PCR. Oh et al. [11] employed RAPD-PCR using the OPR13 primer for typing of *Bacillus* isolates. Their *B. cereus* strains were classified into 19 banding patterns. They reported that the RAPD patterns obtained with OPR13 distinguished better than OPA3. RAPD and Pulsed-Field Gel Electrophoresis (PFGE) were used to assess the similarity between emetic *B. cereus* strains. Seventeen (17) distinct banding patterns were obtained, while 10 strains did not give any banding pattern with PFGE [27]. Also, in this research, five strains SW_79_, MP_117_, WR_123_, RB_155_, and SG_166_ did not give any bands, but the ImageLab software was able to detect bands for MP_117_ and SG_166_. Strains WR_123_ and SW_79_ did not produce any band based on the analysis, while the remaining three clustered very close to them. Ghelradi et al. [28] also used RAPD-PCR to identify *B. cereus* to the strain level and concluded that the method is useful in describing intraspecific changes among organisms.

### 3.2. 16S rDNA Identification of *B. cereus*

The universal couple of primers F1/R2 was able to detect the 16S region of the isolates with a molecular size of 1500 bp. The Basic Local Alignment Search Tool (BLAST) result that has the highest similarity with the biological sequence in the National Centre for Biotechnology Information (NCBI) database was recorded as the identity of the isolates. The sequences deposited in GenBank with their accession numbers and source of each isolate are presented in Table 1. The isolates were identified as *Bacillus cereus*. Oh et al. used the universal primer F27/R1492 and reported that their strains have high similarity with members of the *B. cereus* group in the GenBank.

### 3.3. Phylogenetic Relationship of *B. cereus*

**Figure 3:**
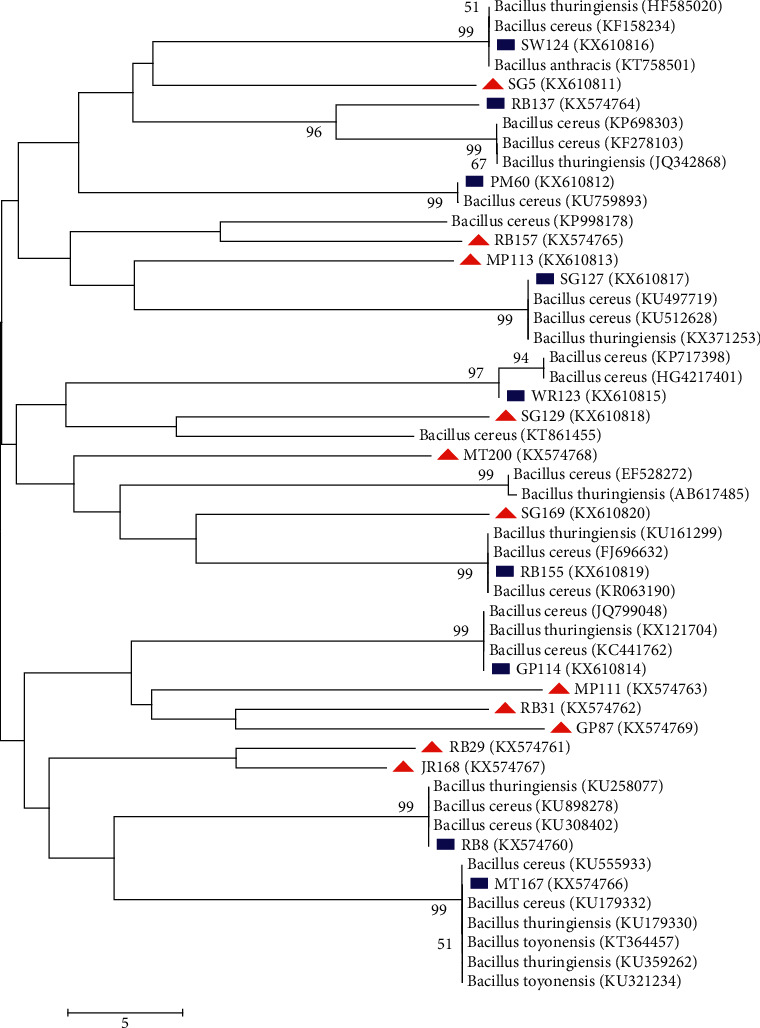
Neighbour-Joining method of phylogenetic tree based on the partial 16S rDNA gene sequence, showing the phylogenetic relationships between the *Bacillus cereus* and the most closely related strains from the GenBank. Sequences obtained in this study higher than 90% are denoted with rectangle and lower than 50% with a triangle.

**Figure 4:**
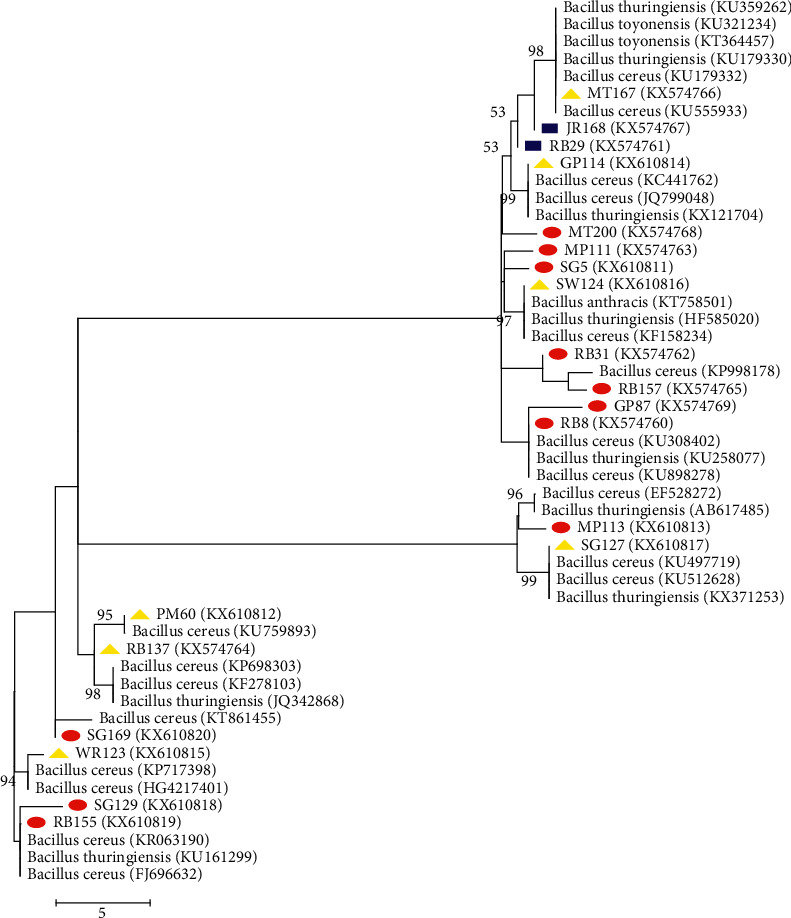
Maximum Likelihood phylogenetic tree based on partial 16S rDNA gene sequence, showing the phylogenetic relationships between the *Bacillus cereus* and the most closely related strains from GenBank. Sequences obtained in this study higher than 90% are denoted with the yellow triangle, greater than 50%, but lower than 80% with the blue rectangle and less than 50% with a red circle.

The phylogenetic relationship between *B. cereus* and the very closely related strains from the GenBank were analyzed using NeighbourJoining (NJ) and MaximumLikelihood (ML) trees (Figures 3 and 4). The percentage of replicate trees in which the associated taxa clustered together in the bootstrap test (1000 replicates) is shown next to the branches. All positions greater than 50% are shown. The two trees were employed to establish the proven resolution and statistical significance of the various treeing algorithms [29, 30]. The NJ tree revealed that RB_8_, PM_60_, GP_114_, WR_123_, SW_124_, SG_127_, RB_137_, RB_155_, and MT_167_ displayed high bootstrap values greater than 90% with their relatives *B. cereus* in the GenBank revealing their similarity percentage. This high bootstrap value expressed by the aforementioned *Bacillus* spp. is beyond 70% borderline of the degree of relatedness proposed by [31]. Furthermore, SG_5_, RB_29_, RB_31_, GP_87_, MP_111_, MP_113_, SG_129_, JR_168,_ and SG_169_ form distinct clades with a bootstrap value lower than 50% but with closest relative to *B. cereus*. They did not cluster with any strains as a result of their nucleotide signature pattern peculiarity, which is in line with the findings of [32]. This distinctiveness calls for their novelty as reported by [30]. This NJ result was based on a cluster-based algorithm utilized in calculating the pairwise distance between sequences and group sequences that are most similar. For clearness, the character-based method (Maximum Likelihood) chooses the best model for the variation pattern of the sequences by correlating a set of data against the set of models of evolution [33]. The ML tree showed that PM_60_, GP_114_, WR_123_, SW_124_, SG_127_, RB_137_, and MT_167_ possessed a very high similarity percentage greater than 90% with *B. cereus* of the reference sequences from the GenBank. This compares well with the result obtained from the NJ with the exception of RB_8_ and RB_155_ that exhibited lower than 50% homology with their *Bacillus* relatives. This may be as a result of mutation.Hence, the relationship between these strains and their *Bacillus* spp. relatives has been wiped out [30]. ML deduced that RB_29_ and JR_168_ have very low similarity percentage with their relative, which is almost the same with the NJ tree. This is not reliable because their DNA reassociation is above the threshold level based on the result depicted by the ML tree [34]. ML tree also showed that some *B. cereus* from this research did not align with any of the reference taxa based on their uniqueness. These include SG_5_, RB_8_, RB_31_, GP_87_, MP_111_, MP_113_, SG_129_, RB_155_, RB_157_, SG_169_, and MT_200_. These eleven isolates are likely to be a far relative of *B. cereus* strains because they possessed distinct nucleotide signature from their relatives in the GenBank.

## 4. Conclusions

This research has shown that some of the isolates are the same as revealed by the dendrogram generated for the RAPD profile. Eleven of the strains form a distinct clade that can never be deduced with conventional methods. The *B. cereus* isolates are associated with foods such as runner bean and green pea, which are used in the preparation of fried rice, while meat pie is one of the snacks consumed by people to satisfy hunger. Extra caution must be taken when preparing foods with these ingredients, and snacks such as meat pie should be consumed when hot to prevent food poisoning.

## Figures and Tables

**Table 1:** *B. cereus* isolates accession number and the source of the samples.

Isolate code	Accession number	Source of food sample	Closest relative	Homology (%)	Access code of the reference strain in the gene bank
RB_8_	KX574760	Runner bean	*Bacillus cereus* strain MS-Deb-PCB-Bac-1	100	MN545143
RB_29_	KX574761	Runner bean	*Bacillus cereus* strain SLG2	97.04	MT043919
RB_31_	KX574762	Runner bean	*Bacillus cereus* strain EG4	99.66	KY435707
MP_111_	KX574763	Meat pie	*Bacillus cereus* isolate HKS 4-2	93.38	DQ289062
RB_137_	KX574764	Runner bean	*Bacillus cereus* strain LCB	94.47	FJ867921
RB_157_	KX574765	Runner bean	*Bacillus cereus* strain XJ22	87.30	KC510021
MT_167_	KX574766	Meat	*Bacillus proteolyticus* strain SUF.04LB	100	MT052616
JR_168_	KX574767	Jollof rice	*Bacillus cereus* strain AMc01NA	97.38	MT052656
MT_200_	KX574768	Meat	*Bacillus cereus* strain BCHA20	88.22	MT533450
GP_87_	KX574769	Green pea	*Bacillus cereus* ATCC 14579	91.34	MT421927
SG_5_	KX610811	Spaghetti	*Bacillus cereus* strain ZMC6	99.41	MN555358
PM_60_	KX610812	Smoked hide	*Bacillus cereus* strain GL34	99.47	MK099886
MP_113_	KX610813	Meat pie	*Bacillus cereus* strain S19	94.58	MN879950
GP_114_	KX610814	Green pea	*Bacillus cereus* strain D21	99.06	KC441762
WR_123_	KX610815	Cooked rice	*Bacillus* sp strain UFSC-40S1	96.51	MT269024
SW_124_	KX610816	Smoked African chad	*Bacillus cereus* strain Ism37	99.75	KP988025
SG_127_	KX610817	Spaghetti	*Bacillus cereus* strain MS-Deb-PCB-Bac-1	100	MN545143
SG_129_	KX610818	Spaghetti	*Bacillus cereus* strain LZH-F21	100	MN099150
RB_155_	KX610819	Runner bean	*Bacillus cereus* strain LE6	99.61	MT279464
SG_169_	KX610820	Spaghetti	*Bacillus cereus* strain BAL34	93.08	KP717398
WR_140_	MT757957	Cooked rice	*Bacillus cereus* strain MD152	100	MT642947
WR_126_	MT757958	Cooked rice	*Bacillus cereus* strain MD152	100	MT642947
WR_88_	MT757959	Cooked rice	*Bacillus cereus* strain S8	100	MT611946
TT_106_	MT757960	Smoked titus	*Bacillus cereus* strain MD152	100	MT642947
WR_13_	MT757961	Cooked rice	*Bacillus cereus* strain MD152	100	MT642947
SG_92_	MT757962	Spaghetti	*Bacillus cereus* strain L2C3	100	MN961108
SG_118_	MT757963	Spaghetti	*Bacillus cereus* strain MD152	100	MT642947
SG_139_	MT772282	Spaghetti	*Bacillus cereus* strain S8	100	MT611946
SG_99_	MT772283	Spaghetti	*Bacillus cereus* strain MD152	99.85	MT642947
RB_84_	MT772284	Runner bean	*Bacillus cereus* strain MD152	100	MT642947

## Data Availability

The data used to support the findings of this study were deposited at NCBI GenBank.
